# Unique Structural Features of Membrane-Bound C-Terminal Domain Motifs Modulate Complexin Inhibitory Function

**DOI:** 10.3389/fnmol.2017.00154

**Published:** 2017-05-24

**Authors:** David Snead, Alex L. Lai, Rachel T. Wragg, Daniel A. Parisotto, Trudy F. Ramlall, Jeremy S. Dittman, Jack H. Freed, David Eliezer

**Affiliations:** ^1^Department of Biochemistry, Weill Cornell Medicine, New YorkNY, United States; ^2^Department of Chemistry and Chemical Biology, Cornell University, IthacaNY, United States

**Keywords:** complexin, amphipathic helix, membrane curvature, ESR, NMR, micelles, synaptic transmission, pi-bulge

## Abstract

Complexin is a small soluble presynaptic protein that interacts with neuronal SNARE proteins in order to regulate synaptic vesicle exocytosis. While the SNARE-binding central helix of complexin is required for both the inhibition of spontaneous fusion and the facilitation of synchronous fusion, the disordered C-terminal domain (CTD) of complexin is specifically required for its inhibitory function. The CTD of worm complexin binds to membranes via two distinct motifs, one of which undergoes a membrane curvature dependent structural transition that is required for efficient inhibition of neurotransmitter release, but the conformations of the membrane-bound motifs remain poorly characterized. Visualizing these conformations is required to clarify the mechanisms by which complexin membrane interactions regulate its function. Here, we employ optical and magnetic resonance spectroscopy to precisely define the boundaries of the two CTD membrane-binding motifs and to characterize their conformations. We show that the curvature dependent amphipathic helical motif features an irregular element of helical structure, likely a pi-bulge, and that this feature is important for complexin inhibitory function *in vivo*.

## Introduction

**FIGURE 1:**
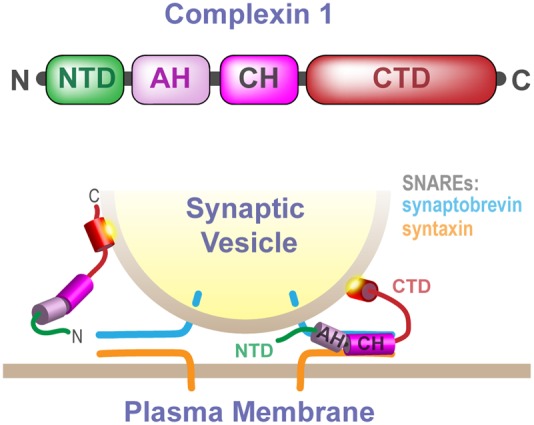
**Cartoon schematic illustrating the different domains of complexin and their interactions with *trans-*SNARE complexes and synaptic vesicles.** Complexin domains are color coded according to the linear representation at the top of the figure, and their interactions are highlighted in the panel below depicting a docked synaptic vesicle anchored to the plasma membrane via the partially zippered *trans-*SNARE complex. The NTD is thought to function in a facilitatory role, the AH contributes to inhibition of vesicle fusion, the central helix binds directly to the SNAREs and is required for all complexin function, and the CTD functions in the inhibition of vesicle exocytosis. Membrane anchoring by two motifs within the complexin CTD, the helical AH motif and the subsequent short unstructured CT motif, localize and position complexin to engage the SNARE complex and inhibit synaptic vesicle fusion.

Synaptic function requires the precise regulation of synaptic vesicle exocytosis by a number of accessory proteins that modulate SNARE complex assembly and synaptic vesicle fusion with the plasma membrane (Rizo and Rosenmund, 2008; Sudhof and Rizo, 2011; Rizo and Xu, 2015; Schneggenburger and Rosenmund, 2015). Complexins are a family of highly conserved cytoplasmic proteins that facilitate calcium-evoked synchronous exocytosis but that can also inhibit spontaneous synaptic vesicle fusion (Brose, 2008; Mohrmann et al., 2015; Trimbuch and Rosenmund, 2016). By clamping synaptic vesicles in a fusion competent but inhibited state until evoked fusion is required, complexins help to prevent premature depletion of the synaptic vesicle pool and keep spontaneous synaptic noise to a minimum (Huntwork and Littleton, 2007; Maximov et al., 2009; Cho et al., 2010; Hobson et al., 2011; Martin et al., 2011; Kaeser-Woo et al., 2012; Wragg et al., 2013). The precise mechanisms by which complexins fulfill two discrete functions remain incompletely delineated. It has been clearly established, however, that complexins contain four domains (**Figure 1**): a highly conserved SNARE-binding central helix necessary for all complexin functions (Pabst et al., 2000; Bracher et al., 2002; Chen et al., 2002; Xue et al., 2007), a facilitatory N-terminal domain (Xue et al., 2007, 2010; Lai et al., 2016), an inhibitory accessory helical domain (Xue et al., 2007; Bin et al., 2010; Krishnakumar et al., 2011; Kümmel et al., 2011; Li et al., 2011; Bykhovskaia et al., 2013; Cho et al., 2014; Radoff et al., 2014; Trimbuch et al., 2014), and an inhibitory C-terminal domain (CTD) (the focus of this work) (Xue et al., 2009; Martin et al., 2011; Wragg et al., 2013; Snead et al., 2014).

The complexin CTD is necessary for the inhibition of spontaneous exocytosis in both worms (Martin et al., 2011; Wragg et al., 2013) and flies (Xue et al., 2009; Cho et al., 2010; Iyer et al., 2013), insofar as the frequency of spontaneous fusion events increases upon deletion or mutation of this domain in these organisms. The function of the mammalian CTD is somewhat more enigmatic, and it has been suggested to both facilitate and inhibit vesicle fusion in different experimental contexts. Mammals contain multiple complexin isoforms with differing CTDs that may contribute in different ways to exocytosis, exerting either a facilitatory or inhibitory effect depending on the specific isoform (Reim et al., 2005; Schaub et al., 2006; Malsam et al., 2009; Seiler et al., 2009; Kaeser-Woo et al., 2012; Yang et al., 2013; Lai et al., 2014). Interestingly, the CTDs of both worm complexin-1 (CPX-1) (Wragg et al., 2013; Snead et al., 2014) and mouse complexin-1 (mCpx1) (Seiler et al., 2009) bind to phospholipid vesicles through a putative amphipathic helical region and in the case of the worm, this membrane interaction is necessary for inhibition of spontaneous synaptic vesicle exocytosis (Wragg et al., 2013; Snead et al., 2014). Fly complexin, too, binds to liposomes *in vitro* (Cho et al., 2015) and contains a putative amphipathic region (Wragg et al., 2013), though whether this region contributes to membrane-binding and protein function in flies remains unknown.

The CPX-1 CTD contains tandem lipid binding motifs – termed the C-terminal (CT) and amphipathic helix (AH) motifs – that together sense membrane curvature to preferentially bind highly curved membranes (Snead et al., 2014). Membrane curvature sensing by these two regions likely directs complexin to the highly curved synaptic vesicle membrane (Snead et al., 2014), its likely membranous binding target *in vivo*. Notably, worm complexin appears to function from the synaptic vesicle, but not the plasma membrane (Wragg et al., 2013). Both motifs are critical for the ability of complexin to inhibit synaptic vesicle exocytosis in worms, as perturbation of either motif impairs inhibitory activity *in vivo*. The CT motif contains a mix of bulky hydrophobic and positively charged residues that likely mediate its interaction with lipid membranes; a CPX-1 variant with this motif deleted cannot bind membranes *in vitro* and cannot inhibit exocytosis *in vivo*. The adjacent amphipathic region is intrinsically disordered in the free state and adopts helical structure only upon binding to highly curved membrane surfaces. Mutations that impair membrane-binding of the amphipathic motif also perturb inhibitory function *in vivo*; interestingly, mutations that selectively decrease helix formation, but not lipid binding, similarly fail to inhibit vesicle exocytosis, suggesting that helix formation by the amphipathic motif is critical for CPX-1 inhibitory function (Snead et al., 2014). Putative amphipathic helical regions have been identified for not only worm but also fly, and mammalian complexins, suggesting that it may be a conserved structural feature of the complexin CTD. In fact, phospholipid membrane-binding by a putative amphipathic helix of the complexin CTD was first established for mammalian complexin-1 (Seiler et al., 2009). Interestingly, many complexins contain a C-terminal CAAX box motif, including mammalian complexin-3 and -4. Farnesylation of this CAAX box motif mediates membrane association for these variants, further arguing that complexin CTD-membrane interactions are of critical functional significance (Brose, 2008; Xue et al., 2009; Cho et al., 2010; Buhl et al., 2013; Iyer et al., 2013).

Why is amphipathic helix formation required for CPX-1 function? To answer this question requires a clearer picture of the structural properties of the membrane-bound CPX-1 CTD. Prior structural characterization of the vesicle-associated CPX-1 CTD using solution-state NMR was limited by the fact that this slowly tumbling high molecular-weight protein–lipid complex is invisible to standard experiments. Optical methods such as circular dichroism (CD) spectroscopy, which are not limited by size, provided some information on helix formation, but only at low resolution (Snead et al., 2014). In this paper, we use two established and complementary techniques to directly observe and so better delineate the structure of the worm complexin-1 CTD in the membrane bound state at a residue-specific level. We first characterize the structure of the CTD in the presence of dodecylphosphocholine (DPC) micelles, a membrane mimetic amenable to study by standard solution-state NMR experiments. We then apply electron spin resonance (ESR) spectroscopy with site-directed spin labeling to directly characterize the AH and CT motifs of phospholipid vesicle bound CPX-1 CTD. Together, these experiments provide a clearer picture of the structure and boundaries of the membrane-binding motifs of the CPX-1 CTD, which is confirmed in an accompanying study (Wragg et al., 2017) using functional assays in living worms. The results provide evidence for an interruption of regular helical structure in the AH motif at residue Gly 116. Deletion of Gly 116 restores regular helical structure to the AH motif and leaves membrane-binding unimpaired, but impairs CPX-1 inhibitory function *in vivo*. We conclude that specific structural features of membrane-bound complexin beyond simple amphipathic helix formation are required for proper inhibitory function and speculate that these act to template functionally important protein–protein interactions. Together with an accompanying study (Wragg et al., 2017), our work suggests that these interactions have undergone divergent evolution, potentially explaining reported differences in the inhibitory activity or nematode and mammalian complexin-1.

## Materials and Methods

### Protein Purification

Worm CPX-1 wild-type full-length (lacking the N-terminal methionine), and the CTD fragment (residues 91–143) were cloned into the pET SUMO vector. Single cysteine mutants were generated via site-directed mutagenesis of the full-length wild-type construct. Δ116 was similarly generated via site-directed mutagenesis of the isolated CTD fragment construct. Proteins were expressed either as previously described (Wragg et al., 2013; Snead et al., 2014) or with direct inoculation of isotopically labeled minimal media. Briefly, BL21(DE3) *Escherichia coli* cells were transformed with the relevant plasmid and grown at 37°C for 5 h in a starter culture of Luria Broth with kanamycin. 2 mL starter culture was then diluted into 100 mL overnight culture – either LB/kanamycin or isotopically labeled minimal media/kanamycin – which was then grown at 37°C for 16–20 h. The overnight culture was diluted to 1 L and grown to an optical density ∼0.6. For unlabeled growths (in LB) and for directly inoculated isotopically labeled growths (in minimal media in either H_2_O or D_2_O containing the appropriate isotopic reagents) protein expression was induced for 3–4 h by addition of IPTG. Alternately, to produce labeled protein as previously described, LB cultures at an optical density of 0.6 were pelleted, washed, and then transferred to isotopically labeled minimal media prior to induction with IPTG. Depending on the specific labeling scheme required, the minimal media used contained the appropriate mixture of 15N-labeled ammonium chloride, ^13^C-labeled glucose, ^13^C^2^H labeled glucose, or and/or ^12^C^2^H labeled glucose in either H_2_O or D_2_O.

Proteins were purified as previously described as well. Briefly, cells were lysed by sonication on ice and clarified by centrifugation at 40,000 rpm for 45 min. SUMO-tagged fusion protein was purified on a Ni-NTA column and then dialyzed into 20 mM Tris pH 8, 150 mM NaCl, 1 mM dithiothreitol. After cleavage by SUMO protease, purified complexin was isolated in the flow-through of a second run over the Ni-NTA column. Protein was dialyzed into distilled water and then frozen and lyophilized.

### Protein Spin Labeling

CPX-1 mutants with single cysteine mutations spanning residues 110–136 were generated as noted above. These proteins were dissolved in PBS (pH 6.1) at 50 μM and mixed with *S*-(2,2,5,5-tetramethyl-2,5-dihydro-1H-pyrrol-3-yl)methyl methanesulfonothioate (MTSL, from Santa Cruz Biotechnology, Dallas, TX, United States) in a 1:10 ratio overnight at 4°C and dialyzed against PBS buffer (pH 6.1) to remove excess MTSL. The spin-labeled proteins were mixed with DOPC:DOPE:DOPS (60:25:15) small unilamellar vesicles (SUVs) at about a 1:200 protein:lipid ratio for 30 min at room temperature in PBS buffer (pH 6.1) before measurements.

### Liposome and Micelle Preparation

Lipids were purchased from Avanti Polar Lipids and were stored at -20°C. Lipids – including DPC – were mixed at the desired ratios and dried under nitrogen gas; residual solvent was then removed by centrifugation under vacuum for 1 h or, for the ESR experiments, in a vacuum drier overnight. The resulting lipid film was then resuspended to the desired stock concentration. Preparation of DPC micelles was complete at this point, and this stock of DPC micelles was mixed with protein for sample preparation. For SUVs, the resuspended lipid film was bath sonicated until the solution became transparent. The SUV solution was then further clarified by ultracentrifugation at 60,000 rpm for 2 h (or, for the ESR experiments, at 13,000 *g* for 10 min). For large unilamellar vesicles (LUVs), the resuspended lipid film was frozen in liquid nitrogen and subsequently thawed in warm water for 10 freeze/thaw cycles. LUVs were then prepared using a 1 mL Avanti Mini-Extruder from Avanti Polar Lipids by extruding 21 times each through 400 nm and then 100 nm pore size polycarbonate films. SUVs and LUVs prepared in this manner typically exhibit a size distribution centered at ∼30 and ∼120 nm diameter, respectively (Snead et al., 2014). SUVs and LUVs were stored at 4°C, while DPC micelles were stored at room temperature; all samples were used within 1 week. Concentration were estimated based on the amount of starting lipid used.

### NMR Spectroscopy

For HSQC experiments, typical spectral widths (ppm) and number of complex data points were 14/1024 and 25/256 for proton and nitrogen, respectively, with some variation from experiment to experiment. Perdeuterated CPX-1 CTD and perdeuterated DPC were used for triple resonance experiments used to assign the CTD in the presence of DPC (^15^N, ^13^C, ^2^H labeled protein) and for the HSQC-NOESY-HSQC experiment for the CTD with DPC micelles (^15^N, ^2^H labeled protein). Complete assignments of resonances observed in the presence of DPC micelles were obtained using the following experiments and parameters: (1) TROSY-based HNCACB – Bruker AVANCE 900 MHz spectrometer equipped with a cryoprobe (New York Structural Biology Center) with spectral widths (ppm) and number of complex data points at 14/1024, 25/64, and 69.9/128 in proton, nitrogen, and carbon dimensions, respectively. (2) TROSY-based HN(CO)CACB – Bruker AVANCE 900 MHz spectrometer equipped with a cryoprobe (New York Structural Biology Center) with spectral widths (ppm) and number of complex data points at 14/1024, 25/64, and 69.9/128 in proton, nitrogen, and carbon dimensions, respectively. (3) TROSY-based HNCACO – Bruker AVANCE 900 MHz spectrometer equipped with a cryoprobe (New York Structural Biology Center) with spectral widths (ppm) and number of complex data points at 14/1024, 25/64, and 15/72 in proton, nitrogen, and carbon dimensions, respectively. (4) TROSY based HNCO – Varian Unity Inova spectrometer equipped with a cryoprobe with spectral widths (ppm) and number of complex data points at 18/1536, 26/44, and 16/128 in proton, nitrogen, and carbon dimensions, respectively. Sequential NOEs for DPC-bound were obtained from an HSQC-NOESY-HSQC experiment with the following parameters – Bruker AVANCE 900 MHz spectrometer equipped with a cryoprobe (New York Structural Biology Center) with spectral widths (ppm) and number of complex data points at 14/1024, 25/72, and 25/72 in proton, nitrogen, and nitrogen dimensions, respectively.

To transfer wild-type CTD/DPC assignments to the Δ116 variant, the following triple resonance experiments were acquired for ^15^N, ^13^C labeled Δ116 CTD in the presence of perdeuterated DPC: (1) HNCA – Bruker AVANCE 900 MHz spectrometer equipped with a cryoprobe (New York Structural Biology Center) with spectral widths (ppm) and number of complex data points at 12/1024, 26/64, and 32/128 in proton, nitrogen, and carbon dimensions, respectively; (2) CBCACONH – Bruker AVANCE 900 MHz spectrometer equipped with a cryoprobe (New York Structural Biology Center) with spectral widths (ppm) and number of complex data points at 12/1024, 26/66, and 70/130 in proton, nitrogen, and carbon dimensions, respectively; (3) HNCO – Bruker AVANCE 700 MHz spectrometer equipped with a cryoprobe (New York Structural Biology Center) with spectral widths (ppm) and number of complex data points at 12/1024, 22/64, and 15/1028 in proton, nitrogen, and carbon dimensions, respectively; (4) HN(CA)CO – Bruker AVANCE 700 MHz spectrometer equipped with a cryoprobe (New York Structural Biology Center) with spectral widths (ppm) and number of complex data points at 12/1024, 22/64, and 15/1028 in proton, nitrogen, and carbon dimensions, respectively.

Resonance assignments for free Δ116 CPX-1 could be reliably transferred for all resonances save those residues in the AH motif proximal to the modification. However, intensity ratio analyses could still be performed for the AH motif as a whole because all shifted but unassigned residues necessarily resided within this motif.

All NMR data were indirectly referenced to 4,4-dimethyl-4-silapentane-1-sulfonic acid and ammonia based on the position of the water resonance. Cα–Cβ secondary shifts were calculated as the difference between the observed carbon chemical shifts and random coil values tabulated from linear hexapeptides in 1 M urea at pH 5.0 and 25°C (Wishart et al., 1995). Amide chemical shift perturbations were calculated as

$$\begin{array}{c} \Delta \delta_{\mathrm{avg}}=\sqrt{\left(1/2)(\Delta \delta_{\mathrm{HN}}^{2}+{\left(\Delta \delta_{N}/5\right)}^{2}\right)} \end{array}$$

where 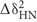 and Δδ_N_ are the amide proton and amide nitrogen chemical shift differences, respectively. Nitrogen shifts are scaled by a factor of five because of intrinsically larger chemical shift values of amide nitrogens.

### Power Saturation ESR

The cw-ESR measurement spectra were collected on an ELEXSYS ESR spectrometer (Bruker Instruments, Billerica, MA, United States) at X-band (9.5 GHz) at RT. The power saturation experiments were performed in air, argon, and 20 mM Ni(II)-diammine-2,2′-(ethane-1,2-diyldiimino) diacetic acid (NiEDDA) with argon conditions. The latter two conditions were achieved by repeatedly degassing and saturating the sample with argon. In each condition, the spectra were recorded as a function of microwave power varied from 0.1 to 200 mW in 30 steps. The number of scans depended on the quality of the signal. The half-saturation parameter (P_1/2_) is obtained by fitting the equation

$$\begin{array}{c} A\;=\;I^{*}\surd \;P^{*}{\left[1+{\left(2^{1/}{}^{\varepsilon }-1\right)}^{*}P/P_{1/2}\right]}^{-\varepsilon }, \end{array}$$

where *P* is the microwave power applied, *A* is the peak-to-peak value of the central line of the spectra, 𝜀 is the line-homogeneity parameter that was obtained from the fitting (we usually obtained 𝜀 = 1.5 for the best fit). The accessibility parameters Π(O_2_) and Π(Ni) are calculated by the equation

$$\begin{array}{c} \begin{array}{c} \Pi (O_{2})\;=\;[P_{1/2}(O_{2})/\Delta H(O_{2})-P_{1/2}(\mathrm{Ar})/\Delta H(\mathrm{Ar})]/\\ \left[P_{1/2}(\mathrm{ref})/\Delta H(\mathrm{ref})],and\Pi (\mathrm{Ni})\;=\;[P_{1/2}(\mathrm{Ni})/\Delta H(\mathrm{Ni}\right)\\ -P_{1/2}(\mathrm{Ar})/\Delta H(\mathrm{Ar})]/[P_{1/2}(\mathrm{Ref})/\Delta H(\mathrm{Ref})], \end{array} \end{array}$$

where *ΔH* is the line width of the central line measured at 2 mW. We used data for Q119C in argon as a reference. The insertion depth parameter Φ, which is independent of the reference, was calculated by the equation

$$\begin{array}{c} \Phi \;=\;ln[\Pi (O_{2})/(\Pi (\mathrm{NiEDDA})](ref1,2,3). \end{array}$$

Experiments were repeated two or three times to ensure reproducibility (Altenbach et al., 1994; Frazier et al., 2002; Georgieva et al., 2014).

The plot of Φ vs. residue number was fit using the equation

$$\begin{array}{c} \Phi \;=\;A^{*}\sin(2\pi^{*}Rn/N+B)+C, \end{array}$$

where *Rn* is the residue number, *N* is the periodicity, *A* is the scaling factor, *B* is the periodicity correction factor, and *C* is the offset. To compare the periodicity of the CTD with a standard alpha helix, we fit the plot in two ways: in the first fitting, *N, A, B*, and *C* were allowed to float; and in the second fitting *N* was fixed at the canonical alpha-helix periodicity of 3.6 residues per turn, and *A, B*, and *C* were allowed to float. The two approaches produced nearly identical fits. Fitting was carried out using the program Origin (Northampton, MA, United States).

### CD Spectroscopy

Far-ultraviolet CD spectroscopy experiments were performed on an AVIV Biomedical Model 410 CD Spectrometer and were performed and analyzed as previously described (Snead et al., 2014). Briefly, spectra were obtained from ∼200 to 250 nm at 25°C with a wavelength step of 1 nm, an averaging time of 1.7 s, 3–4 averaged scans per sample, a cell path length of 0.02 cm, and protein concentrations typically from 50 to 100 uM. CD data are normalized so that the number of helical residues in the absence of lipids matches the predicted free state helicity as estimated by NMR data, as previously described (Snead et al., 2014). Briefly, Cα–Cβ secondary chemical shifts have been tabulated for each amino acid in the presence of fully formed helical structure. We can thus integrate the positive Cα–Cβ secondary chemical shifts for free CPX-1 and normalize by the expected average secondary shift for a fully formed helix to estimate the overall fraction of helicity and the number of helical residues in the free state. By this analysis, the wild-type CTD construct should have roughly 12 residues helical in the absence of liposomes. Once the CD data are properly normalized in the absence of liposomes, the number of helical residues in the presence of lipids is determined using the equation

$$\begin{array}{c} f_{h}=\frac{\left({\left[\theta \right]}_{222}-3000\right)}{-39000}, \end{array}$$

where f_h_ is the fractional helicity, and [𝜃]_222_ is the mean residue ellipticity at 222 nm.

### Lipid Binding Data Analysis

Liposome binding of CPX-1 and CPX-1 mutants and/or truncations was assessed by solution-state NMR spectroscopy, as previously described (Snead et al., 2014). Briefly, HSQC spectra were obtained in the presence and absence of liposomes, and per-residue intensity ratios were calculated as cross-peak intensity with liposomes divided by cross-peak intensity in the free state. Intensity ratios directly reflect the unbound fraction for each residue (Bodner et al., 2009). To generate binding curves for the AH and CT motifs, intensity ratios for residues 111–136 (AH motif) and 137–143 (CT motif) were averaged, and fraction bound was calculated as 1 minus the intensity ratio. As noted above, full assignments were not obtained for free Δ116 CPX-1, though all shifted and unassigned peaks for this variant were necessarily located within the AH motif and so could be incorporated into an average intensity ratio for this motif.

### Strains

*Caenorhabditis elegans* were maintained on agar nematode growth media (NGM) at 20°C and seeded with OP50 bacteria as previously described (Brenner, 1974). Strains employed in this study are *N2, cpx-1 (ok1552), tauIs90* (P*snb-1*::CPX-1::GFP); *cpx-1*, and *tauEx454* [P*snb-1*::CPX-1(ΔG116)::GFP]; *cpx-1*. Robust synaptic expression of all arrays was verified by measuring synaptic fluorescence to check expression levels against those that can fully rescue complexin mutants as previously described (Martin et al., 2011; Wragg et al., 2013).

### Pharmacological Assays

To measure aldicarb sensitivity, 20–25 young adult animals were placed on agar plates containing 1 mM aldicarb (Chem Services) and scored for paralysis at 10 min intervals for 2 h. Each genotype was tested 8–10 times and paralysis curves were generated by averaging paralysis time courses for each plate as described previously (Dittman and Kaplan, 2008; Martin et al., 2011; Wragg et al., 2013).

## Results

### Use of DPC-Micelles to Mimic the Vesicle-Bound State of CPX-1 CTD

**FIGURE 2:**
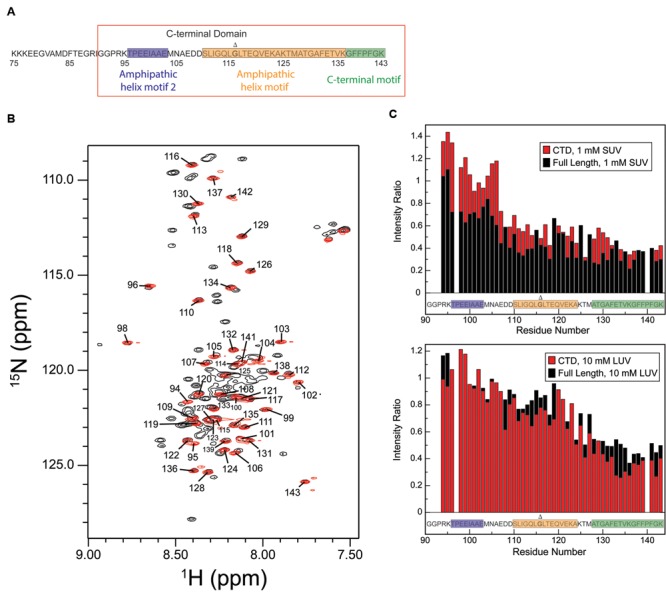
**The CPX-1 CTD behaves similarly when truncated as when in the context of full-length CPX-1. (A)** Sequence of the CPX-1 CTD, highlighting the proposed amphipathic and C-terminal motifs as demarcated in the current study. The CTD construct used throughout this paper is boxed in red. **(B)** Overlay of the assigned HSQC spectrum of free-state full-length CPX-1 (black) with the HSQC spectrum of the isolated C-terminal domain (CTD) (red), also in the free state, demonstrating overlap of the signals. **(C)** NMR intensity ratios observed in the presence of liposomes (top panel SUVs, bottom panel LUVs) versus in their absence for the C-terminal residues of full-length CPX-1 (black) overlaid with intensity ratios observed for the CTD construct (red). Shaded sequences indicate the amphipathic and C-terminal motif sequences as originally defined (Snead et al., 2014). Data for full-length complexin are those shown previously for SUVs (top panel, black data) and those collected for the previously reported LUV binding curves for LUVs (bottom panel, black data) (Snead et al., 2014). Membrane-binding to both SUVs (top) and to LUVs (bottom) by the isolated CTD is comparable to that observed for the full-length protein aside from SUV binding in the region of the AH2 motif, where binding by the truncated CTD is decreased. Gly116, deletion of which is discussed later in the text, is shown in bold and marked with a Δ.

Detergent and phospholipid micelles provide a membrane-like environment yet are small enough for high-resolution structural analysis by NMR, and are therefore often utilized as membrane mimetics. Micelles have proven particularly useful for the structural characterization of peripheral membrane-interactions by proteins such as alpha-synuclein and tau (Eliezer et al., 2001; Bussell and Eliezer, 2003; Barré and Eliezer, 2006; Borbat et al., 2006; Georgieva et al., 2008, 2010), among others. To facilitate solution-state NMR studies of the membrane-binding AH and CT motifs of CPX-1, we produced a recombinant CTD fragment lacking the N-terminal domain, accessory domain, or central helix (**Figure 2A**) in order to reduce spectral complexity and overlap. Proton-nitrogen correlation spectra (HSQC) of the truncated CTD construct free in solution overlay well with the subset of signals in the corresponding spectrum of the full-length protein (**Figure 2B**). Specifically, each peak in the spectrum of the truncated CTD (red signals in **Figure 2B**) is situated at a position that can be unambiguously identified as belonging to an assigned resonance in the corresponding spectrum of the full-length protein (black signals in **Figure 2B**), allowing us to transfer the previously published backbone assignments from the full-length protein to the truncated CTD.

To assess whether the isolated CTD binds to lipids in a manner similar to that observed in the context of the full-length protein, we obtained HSQC spectra in the presence of lipid vesicles. Resonances corresponding to residues that engage in interactions with the vesicle surface are undetectable in such spectra, which therefore provide, for each residue, a measure of the fraction of protein for which that residue is bound to the vesicle surface. In other words, a decrease in the intensity ratio (±lipid) indicates an interaction with the vesicle. The resulting data shows that the AH and CT motifs in the isolated CTD bind to phospholipid vesicles in a manner similar to that previously observed in the context of full-length CPX-1 (**Figure 2C**). Specifically, the top panel shows strong binding to highly curved SUVs for residues 110–143, containing the AH and CT motifs, as previously reported (Snead et al., 2014), which is comparable for the full-length protein and the truncated CTD. Weaker binding is observed for residues 94–107, containing the AH2 motif, as previously observed, but in this region binding of the truncated CTD is decreased compared to the full-length protein. Importantly this effect is well-separated from the AH and CT motifs. The bottom panel shows that a greater concentration of less highly curved LUV vs. more highly curved SUV membranes is required to achieve comparable binding at the very C-terminus for both constructs, in keeping with the established curvature dependence of the CTD interaction. As previously noted (Snead et al., 2014), binding to LUVs decreases moving away from the CT motif to the AH and AH2 motifs. LUV binding by the truncated CTD is very similar to that observed for the full-length protein.

**FIGURE 3:**
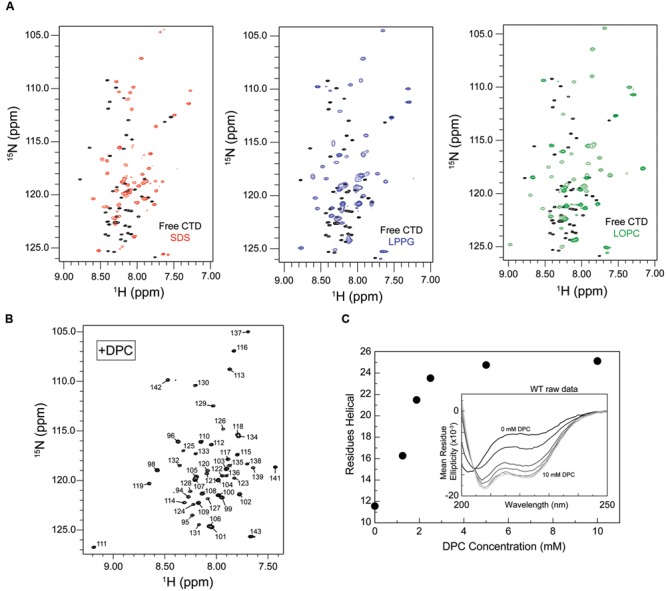
**Increased proton dispersion and CD signals typical of helix formation suggest a disorder-to-order transition of the isolated CTD upon micelle binding. (A)** Unassigned HSQC spectra for the isolated CTD in the presence of 100 mM sodium dodecyl sulfate (SDS, left, red), 50 mM 1-palmitoyl-2-hydroxy-sn-glycero-3-phospho(1′-rac-glycerol) (LPPG, middle, blue), 50 mM 1-oleoyl-2-hydroxy-sn-glycero-3-phosphocholine (LOPC, right, green) superposed on the spectrum of the free state (black). Increased proton and nitrogen dispersion is observed upon addition of any of these three detergents/phospholipids consistent with some degree of structure formation upon micelle binding. **(B)** Assigned HSQC spectrum for the CTD in the presence of ∼50 mM dodecylphosphocholine (DPC). Increased proton dispersion in the presence of DPC micelles (relative to that seen for the free CTD) is consistent with some degree of structure formation upon DPC binding. For resonance assignments, see BMRB entry 27107. **(C)** Helix formation by the truncated CTD as a function of increasing DPC concentration monitored by circular dichroism (CD) spectroscopy. The number of helical residues was estimated based on [𝜃]_222_ as described in methods. Inset: CD spectra for the isolated CTD in the presence of increasing amounts of DPC (spectra shown from black to increasingly lighter shades of gray correspond, respectively, to: 0, 1.25, 1.875, 2.5, 5, and 10 mM). Protein concentration was ∼100 uM.

To identify a suitable micelle system for characterizing CTD binding, HSQC spectra were acquired in the presence of micelles composed of SDS, DPC, and the lysophospholipids LPPG and LOPC. HSQC spectra of the free CTD exhibit a poor dispersion of amide proton resonances, with many signals clustered together and in many cases overlapping (**Figure 2B**), consistent with a predominantly disordered polypeptide in which individual residues are highly dynamic and experience a similar average local environment. Interestingly, the presence of SDS, LPPG, LOPC, or DPC micelles results in chemical shift changes and a somewhat increased dispersion, with the amide proton signals spread over a wider spectral region and better resolved (**Figures 3A,B** and **Supplementary Figure S1**), suggesting some ordering of the polypeptide chain upon micelle binding. Samples prepared using DPC gave the best spectral quality, and this lipid was therefore selected for subsequent structural studies.

To assess the resemblance of the micelle- and vesicle-bound states, we used CD spectroscopy to monitor secondary structure formation. Upon addition of increasing amounts of DPC, CD spectra of the CPX-1 CTD exhibited a clear dose dependent increase in CD spectral signatures indicative of helical secondary structure formation, including minima at 208 and 222 nm (**Figure 3C**). Importantly, the overall gain in helical structure, corresponding to approximately 13 residues, closely matched that observed previously upon SUV binding (Snead et al., 2014), suggesting that the micelle- bound state may accurately represent the more physiologically relevant SUV-bound state.

### Structural Features of DPC-Micelle Bound CPX-1 CTD

Based on liposome binding profiles such as those in **Figure 1C**, we postulated previously (Snead et al., 2014) the existence of three structurally distinct motifs within the CPX-1 CTD: (1) the CT motif spanning residues 128–143 containing multiple bulky hydrophobic phenylalanine residues; (2) the AH motif spanning residues 110–124 that is unstructured in isolation and when bound to flat membranes, but that adopts helical structure upon binding to highly curved membranes; and (3) an AH2 (amphipathic helix 2) motif from residues 96–103 that forms a stable helix even in the free state, that may interact with membranes, and that, like the C-terminal and amphipathic motifs, is necessary for CPX-1 inhibitory function (perturbation of this motif impairs CPX-1 function *in vivo*).

**FIGURE 4:**
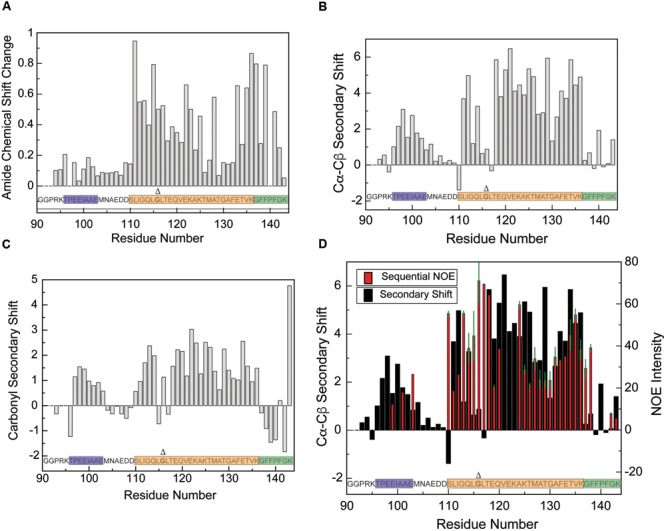
**Structural characterization of the CTD in the presence of DPC micelles by NMR. (A)** Differences of amide proton/nitrogen chemical shifts observed for the CTD in the presence of DPC micelles versus in the free state (absence of micelles), reflecting changes that occur upon micelle binding. Deviations of the Cα–Cβ **(B)** and CO **(C)** chemical shifts observed for the CTD in the presence of DPC micelles from tabulated values typical of random-coil conformations (so-called secondary chemical shifts), reflecting the secondary structure of the micelle-bound state. Positive Cα–Cβ and CO secondary shifts are indicative of helical structure. **(D)** Overlay of Cα–Cβ secondary shift data (black) with sequential amide proton NOE intensities (red) for the CTD in the presence of DPC micelles. The NOE data are plotted such that data at a given residue represents NOEs between that residue and its preceding residue. The average of the forward and reverse NOE is shown; error bars (in green) represent the difference between the two intensities. Data points with no error bars represent peaks for which only one clear NOE cross peak could be observed (i.e., with no symmetric crosspeak), either because the symmetric peak was too weak, or because of spectral overlap. Similarly, blank data points represent residues for which no crosspeaks were observed, either because there was no peak visible or because of spectral overlap. DPC concentration was ∼55 mM and Protein concentrations varied from ∼100 uM for HSQC experiments to ∼500 uM for NOESY experiments. Lipid-to-protein ratios were all above the ∼100:1 saturation point indicated in the CD data. Gly116, deletion of which is discussed later in the text, is shown in bold and marked with a Δ.

Unlike the liposome-bound state, which cannot be visualized directly using solution-state NMR, the DPC micelle-bound state allowed us to directly establish the structure and boundaries of the CTD in a bound state. A standard set of triple resonance spectra acquired using perdeuterated CTD in the presence of perdeuterated DPC micelles enabled complete NMR backbone and C_β_ resonance to be obtained (**Figure 3B**). Comparing the amide group chemical shifts to those of the free CTD in solution (**Figure 4A**) reveals large differences for signals originating from residues 110–143, likely indicating direct protein/micelle interactions in this region, and consistent with the previously proposed location of the AH and CT motifs. Considerably smaller differences are observed for signals corresponding to the N-terminal portion of this construct (residues ∼90–110), suggesting that this region may not interact with micelles. These smaller changes are likely caused by differences in the conditions under which the free and bound states are prepared.

Carbon chemical shifts are sensitive to local secondary structure, which leads to deviations (secondary shifts) from tabulated shifts observed in model random coil peptides (Wishart et al., 1995). The Cα–Cβ secondary shifts observed for the micelle-bound CTD (**Figure 4B**) feature two distinct regions, each exhibiting a nearly contiguous stretch of positive values, while the remaining residues mostly feature smaller values near zero with only isolated exceptions. Residues 96–104 exhibit positive secondary shifts indicative of marginally stable helical structure (average value of 1.8 ± 0.3) and subsume the location of the previously noted AH2 motif. CO secondary shifts (**Figure 4C**) are also positive for residues 96–103 with values consistent with partly populated helical structure. Notably, similar Cα–Cβ secondary shifts were observed in this region for the free protein (Snead et al., 2014). When combined with the lack of micelle-induced amide group chemical shift changes in this region, this suggests that the AH2 motif is marginally stable independently of the presence of DPC micelles, that it does not interact directly with the micelles and that its secondary structure is not influenced by micelle binding.

Larger positive Cα–Cβ secondary shifts (average 4.2 ± 0.3) are observed for residues 111–114 and 118–136, indicating the presence of stable helical structure in these regions. Although the free state of the protein exhibits positive Cα–Cβ secondary shifts for residues 109–126 (Snead et al., 2014), they are of significantly smaller amplitude (average of 0.9 ± 0.4), indicating that while this region transiently samples helical structure in the free state, stable helix formation depends on binding to the micelle. Positive CO secondary shifts are also observed for residues 110–114 and 118–136 consistent with helix formation in the micelle-bound state. Interestingly, residues 115–117, which connect two regions with large positive secondary shifts, exhibit small or negative Cα–Cβ and oscillating CO secondary shifts, suggesting that they may populate non-helical phi-psi peptide bond angles.

Residues 137–143 exhibit mostly small Cα–Cβ secondary shifts that oscillate around zero, but CO secondary shifts are somewhat negative in this region, perhaps indicating a weak preference for extended conformations. Although the significance of this observation is unclear, molecular dynamics simulations in an accompanying paper (Wragg et al., 2017) support a somewhat extended conformation for this region of the protein when bound to lipid membranes.

To further assess the conformation of these different CTD regions when bound to micelles, we measured NH-NH NOEs (Nuclear Overhauser Effects) between neighboring residues. These signals, which reflect inter-nuclear distances between the amide protons of successive residues, are strong in helical regions and weaker in regions of random coil or extended structure. NOEs were detected almost exclusively in regions exhibiting chemical shifts indicative of helical structure (**Figure 4D**), including a few scattered NOEs in the region corresponding to the AH2 motif, and strong NOEs from residues 110–136. Interestingly, residues 115–117 exhibit strong NOEs, indicating short NH-NH distances, despite having secondary shifts that are not typical of helical structure. As discussed below, this region of the protein likely adopts a pi-bulge conformation.

### Secondary Structure of the AH Motif Bound to Lipid Vesicles

Although our CD data suggest that the micelle-bound CTD resembles the vesicle-bound protein, it remains possible that micelles engender a conformation of the protein that is not physiologically relevant. To validate the NMR analysis of the micelle-bound protein, we employed ESR spectroscopy combined with site-directed spin-labeling to probe the environment of each residue in a region encompassing that for which strong NOEs were observed (residues 110–136). Continuous wave ESR spectra were obtained for full-length CPX-1 labeled at each position with MTSL [*S*-(1-oxyl-2,2,5,5-tetramethyl-2,5-dihydro-1H-pyrrol-3-yl) methyl metha- nesulfonothioate] in the presence of SUVs and either O_2_, which selectively partitions into the membrane, or NiEDDA, which partitions into solution. For each residue, MTSL accessibilities to O_2_ (membrane probe) and NiEDDA (solution probe) were determined and used to calculate the membrane insertion depth parameter Phi. Secondary structure results in a regular periodicity of Phi as a function of residue number (Hubbell et al., 1998; Georgieva et al., 2014). SUVs composed of 60:25:15 DOPC:DOPE:DOPS were employed in order to approximate the lipid composition and size (∼30 nm diameter) of synaptic vesicles, as previously described (Snead et al., 2014).

**FIGURE 5:**
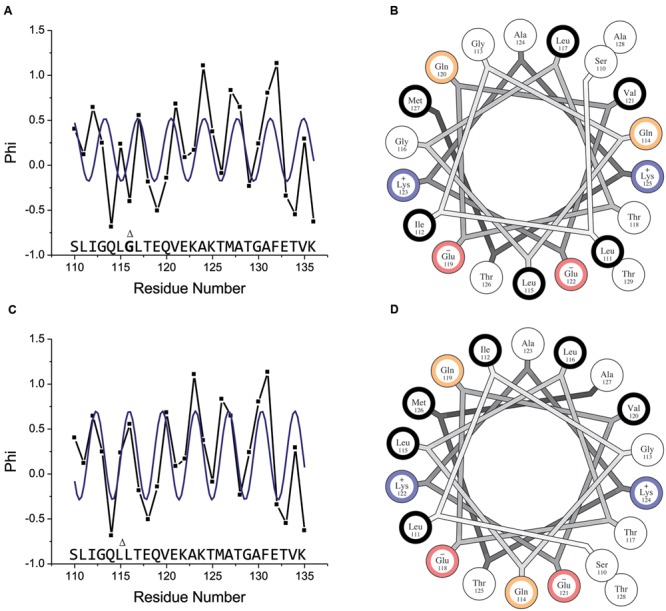
**Membrane-bound helical periodicity of the amphipathic motif is altered around glycine 116. (A)** Continuous-wave EPR analysis of full-length wild-type CPX-1 singly labeled with MTSL at residues 110–136. Shown is the insertion depth parameter Φ, related to the ratio of accessibility to O2 and to NiEDDA as Φ = ln(ΠO2/ΠNiEDDA). Increasing Φ values indicate deeper immersion into the membrane. The blue line indicates the best fit to a cosine function with a fixed periodicity of 3.6 amino acids per turn (i.e., that expected for an ideal alpha helix). **(B)** Helical wheel diagram for the proposed amphipathic region of wild-type CPX-1. Residues are color coded according to their hydrophobicity as hydrophobic (black), neutral (white), polar (yellow), negatively charged (red), and positively charged (blue). The wild-type sequence does not appear amphipathic when modeled as a canonical alpha-helix. **(C)** Same data as shown in **(A)** but with residue 116 omitted. The blue line again indicates the best fit to a cosine function with a fixed periodicity of 3.6 amino acids per turn. **(D)** Helical wheel diagram for the proposed amphipathic region of the CTD with glycine 116 omitted. Removal of this residue results in a clear amphipathic character. Helical wheel plots were generated using the HELNET program suite (Jones et al., 1992). Gly116 is shown in bold and marked with a Δ, which also marks its location when omitted.

For residues 117–136, the ESR data (**Figure 5A**) show a clear periodicity that matches that 3.6 residue per turn periodicity expected for an ideal alpha helix reasonably well. However, this periodicity is not maintained through residues 115–116, where interruptions in the secondary chemical shift data were observed, confirming that regular helical structure is interrupted in this region. Residues 110–114 exhibit periodicity that is again consistent with helical structure. Thus, the observed periodicity deviates from that expected for a canonical alpha helix in a short region centered on residue 116, while the two sequences to either side of this residue appear to adopt regular alpha-helical structure in the bound state. Importantly, the fact that both the ESR (**Figure 5A**) and NMR (**Figure 4**) data indicate an interruption in regular helical structure around position 116 alleviates any concern that the introduced spin label may somehow cause the anomalous behavior.

An explanation for the interruption of regular helical structure around residue 116 can be obtained by plotting the sequence of CPX-1 CTD starting at residue 110 as an helical wheel projection (**Figure 5B**). The resulting helix has hydrophobic residues positioned all around the helix axis. Such a helix does not possess the amphipathic character that is typically seen in helices that bind to membrane surfaces. Closer examination of the helical wheel reveals that residues 110–116 maintain an amphipathic character by clustering hydrophobic residues Leu111, Ile112, and Leu115 opposite from polar/small residues Ser110, Gly113, Gln114, and Gly 116. The next residue in the sequence, however, Leu 117, falls in the middle of the apolar cluster of residues, disrupting the amphipathic moment of the helix.

The same phenomenon is observed by examining a helical wheel projection of the CTD sequence starting at position 116. This results in an amphipathic helix with well-defined polar and apolar faces (**Supplementary Figure S2A**), as shown previously (Wragg et al., 2013) and consistent with the NMR chemical shift data. Attempting to extend this helix in the N-terminal direction, however, results in Leu115 falling in the middle of the polar face of the helix (**Supplementary Figure S2B**), suggesting that a structural rearrangement is required to generate a continuous amphipathic character.

### Deletion of Gly116 Results in a Single Continuous Amphipathic Helix without Altering Membrane-Binding or Curvature Sensing by the AH Motif

**FIGURE 6:**
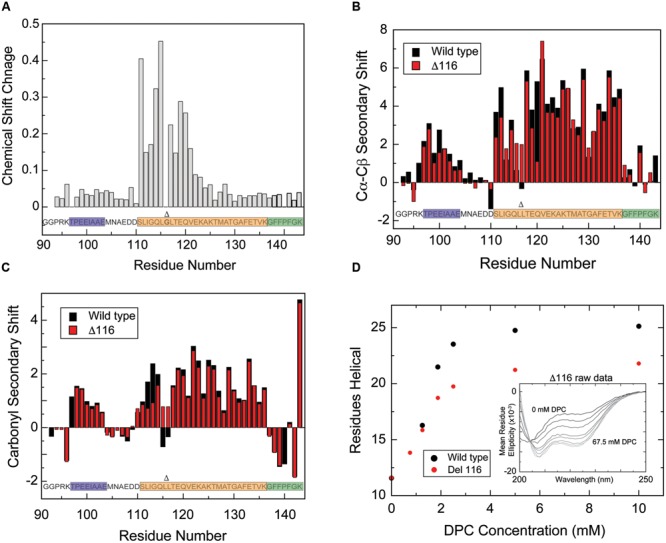
**Deletion of glycine 116 alters the helical structure of the amphipathic motif, though overall helicity is maintained. (A)** Amide proton/nitrogen chemical shift differences for wild-type versus Δ116 CTD. Amide shift perturbations are localized to the area of the deletion. Overlay of Cα–Cβ secondary shifts **(B)** and CO secondary shifts **(C)** for wild-type (black) versus Δ116 (red) CTD in the presence of DPC micelles. Δ116 complexin shows moderately positive Cα–Cβ secondary shifts at residues 115 and 117, while near zero values are observed for wild-type complexin for residues 115–117, suggesting that deletion of residue 116 allows helical structure to propagate through this region. CO secondary shifts also exhibit a clear change toward values consistent with helical structure for residues 115 and 117. Note that for ease of comparison with the Δ116 variant, data for residue 116 of wild-type complexin is not shown. **(D)** Helix formation by wild-type (black) versus Δ116 (red) CTD as a function of increasing DPC concentration monitored by CD spectroscopy. The number of helical residues was estimated based on [𝜃]_222_ as described in methods. Inset: CD spectra for the isolated CTD of Δ116 complexin in the presence of increasing amounts of DPC (spectra shown from black to increasingly lighter shades of gray correspond, respectively, to: 0, 0.75, 1.25, 1.875, 2.5, 5, 10, and 67.5 mM). Gly116 is shown in bold and marked with a Δ, which also marks its location when deleted.

Based on the observation that Gly116 effectively leads to a discontinuity in the amphipathic moment of the helical structure of CPX-1 CTD, we hypothesized that removal of this residue might result in a single uninterrupted amphipathic helix extending from residue 110–136. This hypothesis was supported by the observation that excision of Gly116 from the ESR data leads to a continuous helical periodicity throughout this region (**Figure 5C**) and that a helical wheel projection of the sequence lacking Gly116 is amphipathic (**Figure 5D**). To directly test the hypothesis, we generated a mutant CTD with residue Gly116 deleted, termed Δ116, and obtained backbone and C_β_ NMR resonance assignments in the presence of DPC. Substantial amide chemical shift changes for DPC-bound Δ116 versus wild-type CTD were observed for ∼residues 111–121, centered on the deletion site, indicating that the mutation only affected local structure, with no impact further toward the N- or C-termini (**Figure 6A**). Carbon secondary shifts (**Figures 6B,C**) indicate that DPC-bound Δ116 does not contain the helical break observed for the wild-type CTD, with Cα–Cβ carbon secondary shifts of ∼2 for Δ116 residues 115 and 117 (compared with values of -0.5 to 1 for wt residues 115–117). Carbonyl secondary shifts, too, are positive for residues 115 and 117 in Δ116, in contrast with the wild-type where they are negative. Thus NMR secondary chemical shifts suggest that whereas micelle-bound wild-type CTD contains a helical break at residues 115–117, Δ116 likely adopts a continuous helical structure extending from residue 110 to 136. CD spectra confirm that Δ116 forms a similar degree of helical structure in the micelle-bound state, as wild-type CTD (**Figure 6D**).

**FIGURE 7:**
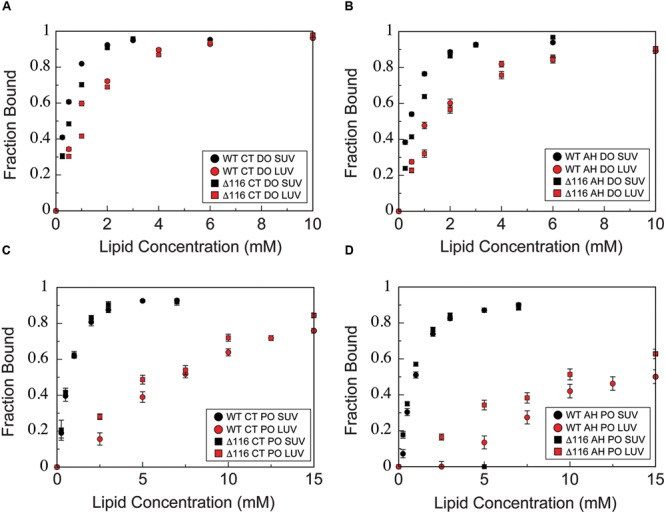
**Membrane-binding is not perturbed by deletion of glycine 116.** NMR-derived binding curves derived as described in methods for **(A)** the CT motif (residues 137–143) in the presence of 60/25/15 DOPC/DOPE/DOPS SUVs (black) and LUVs (red) for the isolated wild-type (circles) and Δ116 (squares) CTD, **(B)** the AH motif (residues 111–136) in the presence of 60/25/15 DOPC/DOPE/DOPS SUVs (black) and LUVs (red) for the isolated wild-type (circles) and Δ116 (squares) CTD, **(C)** the CT motif (residues 137–143) in the presence of 85/15 POPC/POPS SUVs (black) and LUVs (red) for the isolated wild-type (circles) and Δ116 (squares) CTD, **(D)** the AH motif (residues 111–136) in the presence of 85/15 POPC/POPS SUVs (black) and LUVs (red) for the isolated wild-type (circles) and Δ116 (squares) CTD. Error bars represent standard error of the mean.

To determine whether deletion of glycine 116 alters phospholipid vesicle binding or membrane curvature sensing by CPX-1, we titrated wild-type and Δ116 CTD with SUVs or LUVs composed of either 85/15 POPC/POPS or 60/25/15 DOPC/DOPE/DOPS and monitored binding using NMR HSQC spectra. Binding curves for the AH and CT motifs were then generated by averaging the data over all residues within each motif (**Figure 7**). For the wild-type protein, as previously reported (Snead et al., 2014), binding of the both the CT and AH motifs is enhanced for SUVs vs. LUVs of similar composition (black vs. red circles, all panels), reflecting the membrane curvature sensitivity of both motifs. In addition, binding of the AH motif is enhanced for liposome composed of lipids with increased acyl chain unsaturation (DO liposomes, upper panels, vs. PO liposomes, lower panels), likely reflecting the role of membrane packing defects in modulating AH motif binding. Interestingly, deletion of Gly116 does not significantly perturb membrane-binding or curvature sensing by the CTD, as binding curves for the AH and CT motifs of Δ116 are similar to those of the wild-type (circles vs. squares, all panels). Both variants display a similar curvature sensitivity in membrane-binding, with preferential binding to small versus large vesicles (black vs. red symbols, all panels). For both wild-type and Δ116, too, increasing acyl chain desaturation (DO versus PO lipids) increases binding to lower curvature membranes (upper panels vs. lower panels). Thus, deletion of residue 116 does not significantly alter binding properties of the CPX-1 CTD.

**FIGURE 8:**
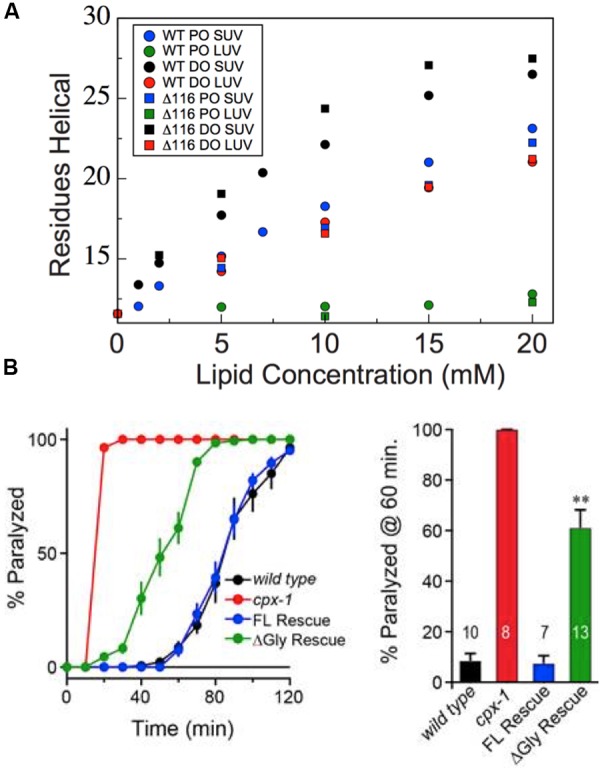
**Deletion of glycine 116 does not perturb membrane-induced helix formation but does impair function *in vivo*. (A)** Helix formation by the truncated CTD of wild-type (circles) and Δ116 (squares) worm CPX-1 upon addition of increasing concentrations of 85/15 POPC/POPS SUVs (blue), 85/15 POPC/POPS LUVs (green), 60/25/15 DOPC/DOPE/DOPS SUVs (black), or 60/25/15 DOPC/DOPE/DOPS LUVs (red) as monitored by CD spectroscopy. Comparable liposome conditions are grouped by color. Data for the wild-type protein are as reported previously (Snead et al., 2014), except that the data for wild-type CTD in the presence of DO SUVs was improperly normalized for protein concentration originally and has been renormalized here. **(B)** Aldicarb assays in wild-type, CPX-1 knockout (*cpx-1*), wild-type full-length rescue (FL), and Δ116 full-length rescue (ΔGly) demonstrating that deletion of Gly116 impairs the ability of CPX-1 to rescue aldicarb induced paralysis. Quantification is shown for paralysis at 60 min on aldicarb with number of trials annotated on each data bar. ^∗∗^Indicates a *p* < 0.01, based on a *post hoc* multi-comparison Tukey–Kramer test done after ANOVA.

To characterize whether deletion of glycine 116 alters helical folding of the AH motif upon liposome binding, we monitored titrations using CD spectroscopy to assess secondary structure (**Figure 8A**). Deletion of glycine 116 does not appear to significantly alter or perturb helical folding by the AH upon binding to liposomes with any of the compositions and sizes tested. The increase upon addition of liposomes in the number of helical residues estimated based on the CD signal at 222 nm is comparable for both wild-type and Δ116 CTD. Together with the above results, these observations suggest that the Δ116 CTD binds to vesicles with a slightly altered bound state structure, but with binding properties and overall helicity otherwise comparable to wild-type CPX-1. Where wild-type CTD contains a helical break in the bound state, Δ116 CTD instead adopts a single continuous helix.

### Irregular Structure at Gly116 Is Necessary for Inhibition of Spontaneous Exocytosis in *C. elegans*

To determine whether altered helical periodicity at residue 116 is important for CPX-1 function, we examined the inhibitory function of wild-type versus Δ116 CPX-1 using a well-established functional assay in *C. elegans* (Miller et al., 1996; Mahoney et al., 2006; Martin et al., 2011). Briefly, upon treatment with the acetylcholinesterase inhibitor aldicarb, wild-type worms paralyze with a characteristic time course. Mutations that increase acetylcholine release at the neuromuscular junction cause worms to paralyze more rapidly. Knockout of *cpx-1* abolishes its inhibition of synaptic vesicle exocytosis, and thus *cpx-1* knockout worms are hypersensitive to aldicarb treatment. This phenotype can then be fully rescued by re-expression of wild-type CPX-1. Interestingly, expression of Δ116 CPX-1 in the *cpx-1* knockout background only partially rescues the aldicarb phenotype (**Figure 8B**), suggesting that the inhibitory function of Δ116 CPX-1 is impaired *in vivo*. Given that Δ116 CPX-1 lacks the helix interruption found in the wild-type, this implies that altered helical periodicity at residue 116 somehow contributes to inhibition of synaptic vesicle exocytosis by CPX-1. Interestingly, the Δ116 mutant is unique in that membrane-binding and overall helicity are not perturbed, though protein function is still impaired. Thus, some property of the CPX-1 CTD beyond membrane-binding and helix formation is likely required for proper inhibitory function.

## Discussion

Membrane-binding by worm CPX-1 via its CTD is required to localize complexin to synaptic vesicles at presynaptic nerve terminals in living worms (Wragg et al., 2013). Membrane-binding requires residues at the very C-terminus of the CTD (CT motif). Furthermore, helix formation by an adjacent amphipathic region (AH motif) upon binding to highly curved membranes is required for efficient inhibition of neurotransmitter release by CPX-1 (Wragg et al., 2013; Snead et al., 2014). A third amphipathic motif (AH2 motif) upstream of the AH motif is also partly helical and important for proper CPX-1 function. The three CTD motifs were defined previously based on NMR measurements of residue-resolved binding of the CTD to lipid vesicles. Here, solution-state NMR and ESR spectroscopy applied to the CPX-1 CTD in the presence of DPC micelles allow us to directly characterize the structural properties of the CTD in a membrane or membrane-like environment while also refining the previously proposed structure and boundaries of these motifs. We find that the AH2 motif, which is marginally stable even in the absence of lipids, is unaffected by the presence of DPC. The CT motif, which is absolutely required for CTD membrane-binding, is found to consist of only the final seven residues, 137–143, of the CPX-1 sequence, and does not adopt regular secondary structure upon binding to micelles. The AH motif is now observed to extend from residue 110 to residue 136, and adopts an amphipathic helix that is interrupted in the region surrounding residue Gly116. Removal of Gly116 results in a continuous helical structure throughout the AH motif, without perturbing membrane-binding or curvature-sensitive helix formation. Despite this, deletion of Gly116 significantly reduces the ability of CPX-1 to inhibit neurotransmitter release in living worms.

### AH2 Motif

The AH2 motif was defined to include residues 96–104 based on the observation that this region possesses amphipathic character, binds to SUVs, albeit more weakly than the CT or AH motifs, and possesses significant helical character even in the free state of CPX-1 (Snead et al., 2014). Surprisingly, we observe no indications of interactions between the AH2 motif and DPC micelles, as neither amide nor carbon chemical shifts are altered in the presence of DPC. This likely results from the choice of our CTD construct, which begins at CPX-1 residue 90, only a few residues away from the beginning of the AH2 motif. We expected that this construct would properly capture the behavior of this motif, but unfortunately it appears that the close proximity of the N-terminus of this fragment may interfere with proper lipid interactions in this region, as observed in our SUV binding data (**Figure 2C**). Obtaining a clearer picture of the structure of the AH2 motif in its lipid-bound state will require a model system that faithfully recapitulates its observed interaction with vesicles.

### CT Motif

We documented the presence of a membrane-binding C-terminal motif in CPX-1 based on the observation that residues at the very C-terminus of CPX-1 bound most tightly to lipid vesicles *in vitro*, combined with the observation that deletion of the final 12 residues of CPX-1 completely abolished membrane-binding by the CTD and significantly impaired inhibitory function *in vivo* (Wragg et al., 2013; Snead et al., 2014). Data presented here suggests, however, that the CT motif likely consists of only the final seven residues of CPX-1, as residues upstream of this region form part of a continuous helical structure that belongs to the adjacent AH motif. Based on small Cα–Cβ secondary chemical shifts that oscillate around zero and on the paucity of sequential amide proton NOEs, we conclude that the CT motif likely binds to micelles in a disordered conformation devoid of secondary structure. This is consistent with previous observations using CD spectroscopy showing no spectral changes upon binding of CPX-1 to LUV liposomes under conditions where the CT motif binds but the AH motif does not become helical (Snead et al., 2014). Membrane-binding by the CT motif is likely mediated by insertion of its three bulky, hydrophobic phenylalanine residues. Based on these new structural data we show in an accompanying paper (Wragg et al., 2017) that mutants in which the final six residues of CPX-1 are deleted, or in which all three CT motif phenylalanine residues are replaced by alanine, bind very weakly to liposomes *in vitro* and are unable to inhibit synaptic vesicle exocytosis in worms. This provides strong support for this new structure-based demarcation of the CT motif. Notably, we previously established that mCpx1 also binds preferentially to highly curved membranes, possibly through tandem lipid binding motifs (Snead et al., 2014) and showed that the putative mCpx1 CT motif consists of seven residues, indicating that the length of the CT motif is likely similar between worms and mice. Despite this similarity, we show in an accompanying paper (Wragg et al., 2017) that the mouse CT motif cannot substitute for the worm motif *in vivo*, suggesting some degree of divergence in the precise function(s) of this motif.

### AH Motif

The AH motif was initially identified by inspection of the CPX-1 sequence (Wragg et al., 2013), though a similar sequence was noted in mammalian complexins by others (Takahashi et al., 1995; Seiler et al., 2009). We confirmed membrane-association of the AH motif using NMR binding assays, and demonstrated that the AH motif becomes helical upon binding to highly curved liposomes using a combination of CD spectroscopy and mutational analysis (Seiler et al., 2009; Wragg et al., 2013; Snead et al., 2014). Here, we directly observe the helical structure of the AH motif when bound to micelles. The motif is found to extend from residues 110 to 136, encompassing a longer stretch of the CPX-1 CTD than was initially proposed based on either sequence analysis (Wragg et al., 2013) or NMR binding assays (Snead et al., 2014). Our secondary chemical shift data indicate, however, that the helical structure of the AH motif deviates from that of a typical alpha helix in the vicinity of residue 116. This deviation can be explained by the fact that the amphipathic moment of the N-terminal part of the AH motif, comprised of residues 110–116, is mismatched with that of the remaining sequence of the motif (residues 117–136), so that formation of a continuous helix throughout the sequence would result in the loss of the overall amphipathic moment. Examination of the sequence reveals that this mismatch effectively results from a single-residue insertion, at position 116, into what would otherwise be a continuous amphipathic helix. Indeed, we show that deletion of Gly116 removes the discontinuity in the AH motif in results in a single continuous helix.

### Pi-bulge in the CPX-1 CTD

**FIGURE 9:**
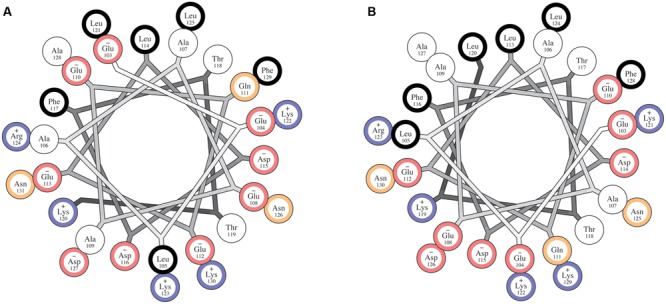
**The CTD of fly complexin may also contain altered helical periodicity in the membrane bound state. (A)** Helical wheel diagram for a proposed amphipathic region of wild-type fly complexin. Residues are color coded according to their hydrophobicity as hydrophobic (black), neutral (white), polar (yellow), negatively charged (red), and positively charged (blue). Similarly to worm CPX-1, the wild-type sequence does not appear amphipathic when modeled as a canonical alpha-helix. **(B)** Helical wheel diagram for the proposed amphipathic region of the fly complexin CTD with glutamate 112 omitted. Removal of this residue clearly increases the amphipathic character of this region. Helical wheel plots were generated using the HELNET program suite (Jones et al., 1992).

The insertion of an extra residue into a helix-forming sequence often results in a rearrangement of the helical turn including that residue into a non-canonical helical structure (Heinz et al., 1993; Keefe et al., 1993). This type of irregularity often takes the form of a pi-helix or a pi-bulge, though such motifs have been referred to also as alpha-bulges or alpha aneurysms, among other names (Keefe et al., 1993; Riek and Graham, 2011). Pi-bulge formation at residue Gly116 is suggested by small, negative and/or oscillating values of Cα–Cβ and carbonyl secondary shifts for residues 115–117, combined with the presence of strong sequential amide proton NOEs through this region. Together these observations indicate a non-canonical helical structure that is nevertheless well-organized and compact, not flexible and disordered. Although our current NMR data are not sufficient to unambiguously establish the presence of a Pi-bulge or to calculate a robust atomic resolution structure, ESR data further corroborated the presence of an irregular helical structure at residue 116; sequences to either side of this residue appear to adopt a canonical alpha helical periodicity that is not propagated through position 116. Interestingly, analysis of the fly complexin sequence, which also contains an AH motif (Wragg et al., 2013; Cho et al., 2015), suggests that the presence of a pi-bulge in the AH motif may be conserved between worms and flies. Similar to worm complexin, residues 103–131 of fly complexin isoform 7B, which, like CPX-1, lacks a C-terminal CAAX box prenylation motif, when modeled using a helical wheel, do not exhibit a clear amphipathic nature (**Figure 9A**). The C-terminal part of this region, starting at residue Glu 112, does form a clearly amphipathic helix (**Supplementary Figure S3A**), but when propagated in the N-terminal direction, residue Gln111 and Glu 110 fall on the hydrophobic face of the helix (**Supplementary Figure S3B**). As in the worm protein, removal of a single residue, Glu112, from the sequence results in a single continuous amphipathic alpha helix (**Figure 9B**). Ongoing efforts to determine structures of both worm and fly complexins in their micelle-bound states should establish the precise nature of their helical structures.

Pi-bulges are often enriched at functional sites within proteins (Cooley et al., 2010) and associated with specific protein functions, for example by acting as a scaffold to align key residues for, e.g., metal ion coordination (Riek and Graham, 2011) or ligand binding (Cartailler and Luecke, 2004). Of greater potential relevance for the case of complexin is the observation that pi-bulges can cause helix bending and helical kinks (Cartailler and Luecke, 2004), structural features which could impact membrane-binding and membrane curvature sensing. To assess whether the presence of a pi-bulge at Gly116 in CPX-1 might contribute to membrane-binding, curvature sensing and inhibitory function, we generated and characterized Δ116 CPX-1. NMR secondary chemical shifts show that deletion of Gly116 removes the pi-bulge, as Δ116 formed a continuous amphipathic helix in the micelle-bound state. Surprisingly, however, membrane-binding, membrane curvature sensitivity, and overall helicity are unperturbed for Δ116. Despite the lack of change in membrane-binding, curvature sensing and helix formation, Δ116 was less able to inhibit neurotransmitter release in *C. elegans*, arguing that the pi-bulge of wild-type complexin contributes to its inhibitory function.

If membrane-binding, curvature sensing and helicity are not impaired, why does Δ116 CPX-1 display decreased inhibitory activity *in vivo*? Though this remains unclear at present, one possibility is that pi-bulge formation contributes to some functionally requisite protein/protein interaction, and removal of Gly116 results in an altered conformation that is unable to properly form this interaction. In an accompanying paper (Wragg et al., 2017) we show that the AH motif of mCpx1 is unable to substitute for the worm AH in CPX-1 despite the fact that the mouse AH motif also binds membranes in a curvature-sensitive fashion (Snead et al., 2014; Gong et al., 2016). We speculate that while membrane-binding and curvature sensing are a requisite conserved features of both AH motifs, additional interactions with unknown binding partners are also required to effect proper complexin function, and that the interactions with these partners, as well as the identity of these partners, may have diverged between worm and mouse, as well as among other phyla. It will thus be critically important to identify any such putative yet currently unknown AH motif protein binding partners.

## Author Contributions

Conceptualization: DS, JD, JF, and DE; methodology: DS, AL, RW, DP, TR, JD, JF, and DE; investigation: DS, AL, RW, DP, TR, and DE; writing: DS, AL, JD, JF, and DE; funding acquisition: DS, DP, JD, JF, and DE.

## Conflict of Interest Statement

The authors declare that the research was conducted in the absence of any commercial or financial relationships that could be construed as a potential conflict of interest.

## Supplementary Material

FIGURE S1 **Dodecylphosphocholine (DPC) micelles lead to increased spectral dispersion in 2D spectra of CPX-1 CTD.** Overlay of HSQC spectra for the CPX-1 CTD in the free state (black) and in the presence of DPC micelles (cyan). Cyan and black horizontal and vertical arrows highlight the increased proton and nitrogen spectral widths spanned by the DPC micelle versus free state spectrum. Inset: expansion of the glycine region similarly shows increased dispersion of these residues.

FIGURE S2 **Propagating the CPX-1 AH motif helix in the N-terminal direction past residue Gly116 results in a loss of amphipathic character. (A)** Helical wheel projection of CPX-1 residues 116–132 shows clearly defined polar and apolar faces. **(B)** Helical wheel projection of CPX-1 residues 115–132 shows that residue Leu115 falls in the middle of the polar face of the otherwise amphipathic helix, disrupting its amphipathic character. Residues are color coded according to their hydrophobicity as hydrophobic (black), neutral (white), polar (yellow), negatively charged (red) and positively charged (blue). Helical wheel plots were generated using the HELNET program suite (Jones et al., 1992).

FIGURE S3 **Propagating the fly complexin isoform 7B AH motif helix in the N-terminal direction past residue Glu112 results in a loss of amphipathic character. (A)** Helical wheel projection of fly 7B complexin residues 112–131 shows clearly defined polar and apolar faces. **(B)** Helical wheel projection of fly 7B complexin residues 110–131 shows that residues Gln111 and Glu110 fall on the apolar face of the otherwise amphipathic helix, disrupting its amphipathic character. Residues are color coded according to their hydrophobicity as hydrophobic (black), neutral (white), polar (yellow), negatively charged (red), and positively charged (blue). Helical wheel plots were generated using the HELNET program suite (Jones et al., 1992).
