# Supplementary figures and images for: Unique Structural Features of Membrane-Bound C-Terminal Domain Motifs Modulate Complexin Inhibitory Function
Source: Front Mol Neurosci. 2017 May 24;10:154. doi: 10.3389/fnmol.2017.00154 (PMC5442187; doi:10.3389/fnmol.2017.00154)

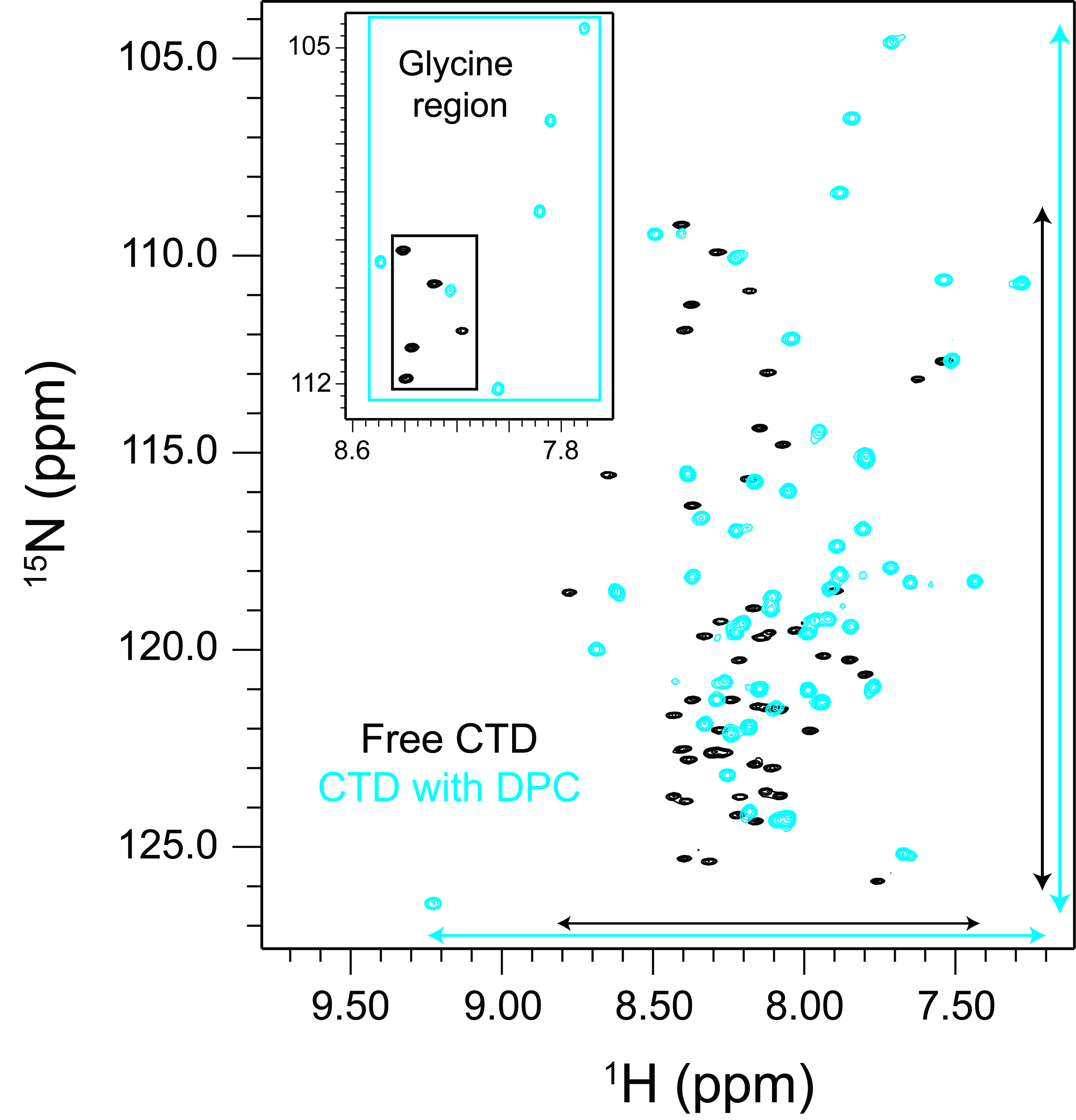

Supplement: FIGURE S1 — Dodecylphosphocholine (DPC) micelles lead to increased spectral dispersion in 2D spectra of CPX-1 CTD. Overlay of HSQC spectra for the CPX-1 CTD in the free state (black) and in the presence of DPC micelles (cyan). Cyan and black horizontal and vertical arrows highlight the increased proton and nitrogen spectral widths spanned by the DPC micelle versus free state spectrum. Inset: expansion of the glycine region similarly shows increased dispersion of these residues. [file Image_1.TIF]

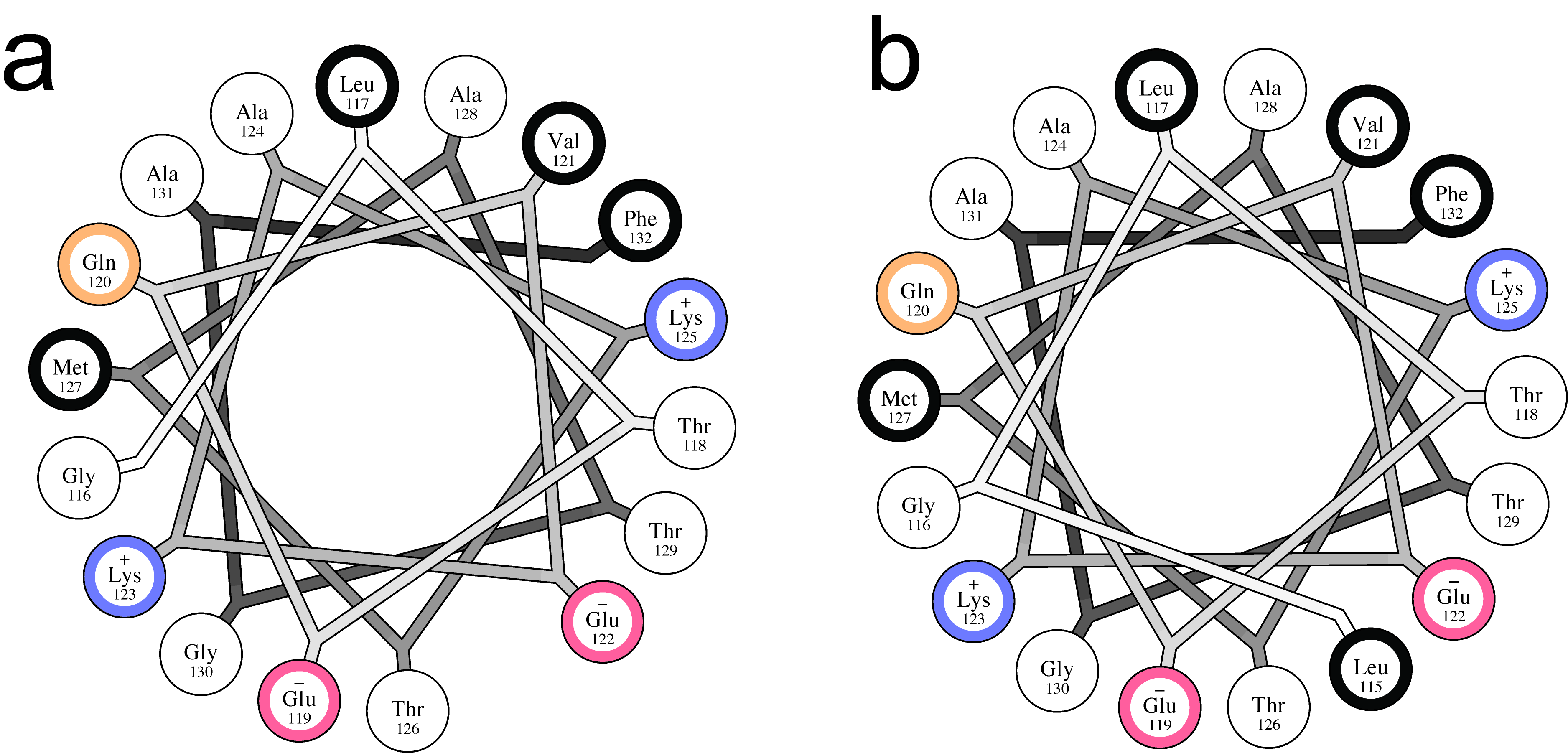

Supplement: FIGURE S2 — Propagating the CPX-1 AH motif helix in the N-terminal direction past residue Gly116 results in a loss of amphipathic character. (A) Helical wheel projection of CPX-1 residues 116–132 shows clearly defined polar and apolar faces. (B) Helical wheel projection of CPX-1 residues 115–132 shows that residue Leu115 falls in the middle of the polar face of the otherwise amphipathic helix, disrupting its amphipathic character. Residues are color coded according to their hydrophobicity as hydrophobic (black), neutral (white), polar (yellow), negatively charged (red) and positively charged (blue). Helical wheel plots were generated using the HELNET program suite (Jones et al., 1992). [file Image_2.TIF]

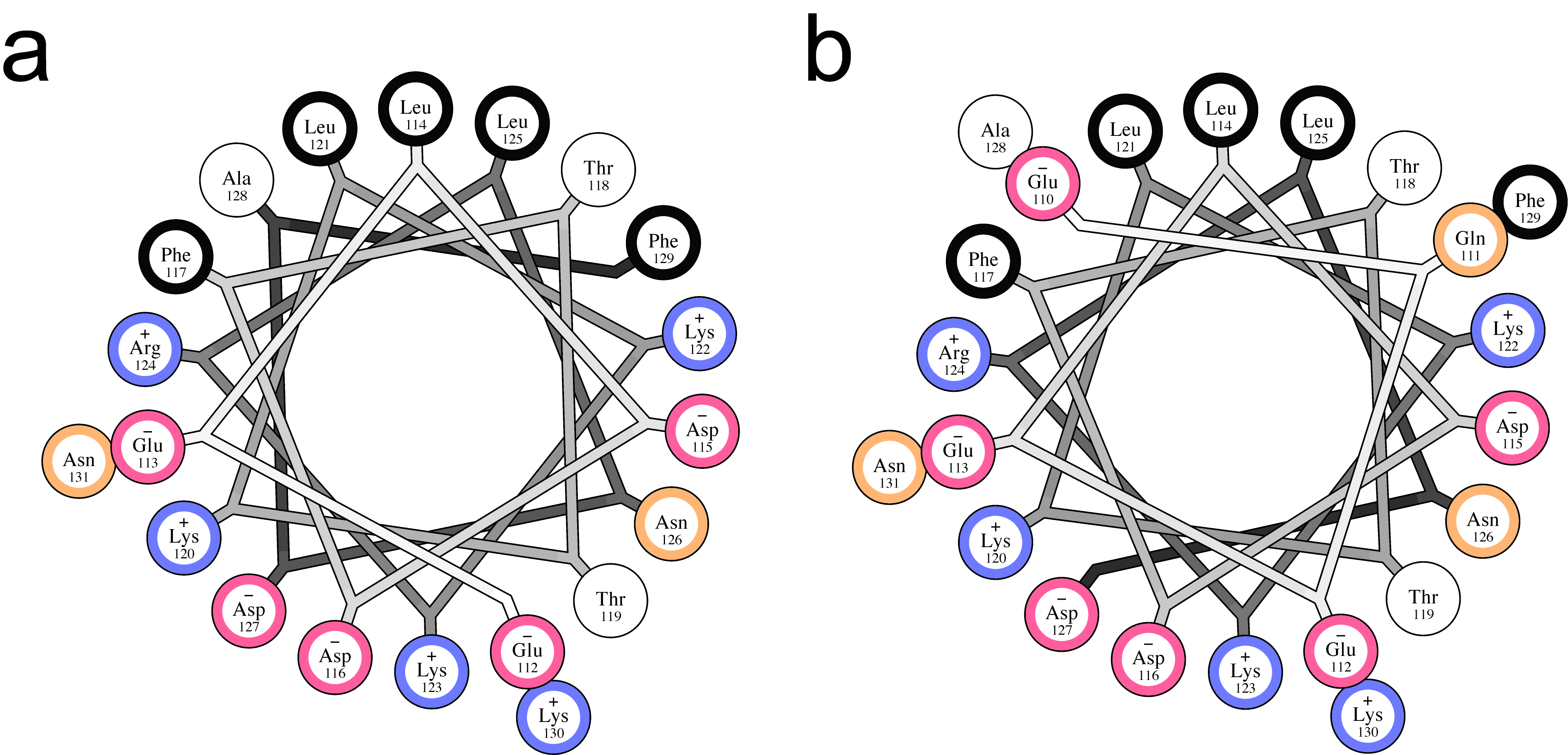

Supplement: FIGURE S3 — Propagating the fly complexin isoform 7B AH motif helix in the N-terminal direction past residue Glu112 results in a loss of amphipathic character. (A) Helical wheel projection of fly 7B complexin residues 112–131 shows clearly defined polar and apolar faces. (B) Helical wheel projection of fly 7B complexin residues 110–131 shows that residues Gln111 and Glu110 fall on the apolar face of the otherwise amphipathic helix, disrupting its amphipathic character. Residues are color coded according to their hydrophobicity as hydrophobic (black), neutral (white), polar (yellow), negatively charged (red), and positively charged (blue). Helical wheel plots were generated using the HELNET program suite (Jones et al., 1992). [file Image_3.TIF]
